# Elevated Expression of Toxin TisB Protects Persister Cells against Ciprofloxacin but Enhances Susceptibility to Mitomycin C

**DOI:** 10.3390/microorganisms9050943

**Published:** 2021-04-27

**Authors:** Daniel Edelmann, Florian H. Leinberger, Nicole E. Schmid, Markus Oberpaul, Till F. Schäberle, Bork A. Berghoff

**Affiliations:** 1 Institute for Microbiology and Molecular Biology, Justus Liebig University Giessen, 35392 Giessen, Germany; Daniel.B.Edelmann@bio.uni-giessen.de (D.E.); Florian.Leinberger@mikro.bio.uni-giessen.de (F.H.L.); Nicole.E.Schmid@mikro.bio.uni-giessen.de (N.E.S.); 2 Branch for Bioresources, Fraunhofer Institute for Molecular Biology and Applied Ecology (IME), 35392 Giessen, Germany; Markus.Oberpaul@ime.fraunhofer.de (M.O.); Till.F.Schaeberle@agrar.uni-giessen.de (T.F.S.); 3 Institute for Insect Biotechnology, Justus Liebig University Giessen, 35392 Giessen, Germany; 4 Partner Site Giessen-Marburg-Langen, German Center for Infection Research (DZIF), 35392 Giessen, Germany

**Keywords:** toxin-antitoxin systems, DNA damage, SOS response, fluoroquinolones, mitomycin C, persistence

## Abstract

Bacterial chromosomes harbor toxin-antitoxin (TA) systems, some of which are implicated in the formation of multidrug-tolerant persister cells. In *Escherichia coli*, toxin TisB from the *tisB*/*istR-1* TA system depolarizes the inner membrane and causes ATP depletion, which presumably favors persister formation. Transcription of *tisB* is induced upon DNA damage due to activation of the SOS response by LexA degradation. Transcriptional activation of *tisB* is counteracted on the post-transcriptional level by structural features of *tisB* mRNA and RNA antitoxin IstR-1. Deletion of the regulatory RNA elements (mutant Δ1-41 Δ*istR*) uncouples TisB expression from LexA-dependent SOS induction and causes a ‘high persistence’ (*hip*) phenotype upon treatment with different antibiotics. Here, we demonstrate by the use of fluorescent reporters that TisB overexpression in mutant Δ1-41 Δ*istR* inhibits cellular processes, including the expression of SOS genes. The failure in SOS gene expression does not affect the *hip* phenotype upon treatment with the fluoroquinolone ciprofloxacin, likely because ATP depletion avoids strong DNA damage. By contrast, Δ1-41 Δ*istR* cells are highly susceptible to the DNA cross-linker mitomycin C, likely because the expression of SOS-dependent repair systems is impeded. Hence, the *hip* phenotype of the mutant is conditional and strongly depends on the DNA-damaging agent.

## 1. Introduction

Bacteria are equipped with numerous systems to sense environmental stress factors and transduce the perceived stress signals into adequate responses. These stress responses aim to repair the stress-induced damages, maintain essential cellular functions, and adjust the physiological status to the stressful situation. However, if stress levels are elevated, regular stress responses might not be sufficient to maintain survival. For such fatal situations, bacteria have evolved survival strategies that are based on the formation of stress-tolerant cells through phenotypic variation [1,2]. The corresponding subpopulations sacrifice their own propagation for the survival of the whole population, thereby increasing the overall fitness of the genotype in unpredictable environments [3].

The formation of stress-tolerant cells is often enhanced or triggered by stress, which also applies to persister cells [4,5,6]. Persister cells were first described in the 1940s [7,8], and are probably present in every bacterial population. They represent phenotypic variants with increased tolerance to high concentrations of stressors, including antibiotics. While an antibiotic rapidly kills the sensitive part of a population, persister cells endure the treatment for a prolonged period. Therefore, the presence of persister cells is revealed by a characteristic biphasic killing curve [4,9]. Even though persister subpopulations are very heterogeneous in terms of persister formation mechanisms [10,11], major cellular processes are typically inhibited in persister cells, resulting in growth retardation and inactivation of antibiotic targets, often referred to as dormancy [6]. However, complete dormancy is not necessary for persister formation, and persister cells might retain metabolic activity [12,13,14]. In *Escherichia coli*, it was even observed that persisters actively extrude β-lactam antibiotics by efflux pumps, rather than rely on passive dormancy for protection [15].

The persistence mechanism might also strongly depend on the specific action of an antibiotic. Quinolone antibiotics, for example, inhibit topoisomerase II (gyrase) and IV, thereby causing double-strand breaks (DSBs). Bacteria respond to DSBs by induction of the SOS response. The RecBCD enzyme complex binds to double-stranded DNA ends and produces single-stranded 3′ tails to enable loading with recombinase RecA, which initiates DSB repair via homologous recombination [16]. RecA-nucleofilaments subsequently trigger self-cleavage of the LexA repressor, leading to activation of the SOS response [17]. LexA-regulated genes contain a 20-bp LexA-box sequence in their promoter regions. The heterology index (HI) indicates the similarity of a particular LexA-box to the consensus sequence [18]. Genes that have a LexA-box with a low HI are tightly repressed by LexA and, hence, strongly induced upon LexA cleavage [19,20,21]. LexA regulates many genes with a function in DNA repair, but also the progression of cell division. For example, SulA inhibits cell division to extend the time that is needed for DNA repair [22]. When *E. coli* cultures are treated with DNA-damaging fluoroquinolones, persistence clearly depends on a functional SOS response, including specific DNA repair proteins [23,24]. Furthermore, SOS pre-induction by low fluoroquinolone levels supports persistence when antibiotic concentrations are subsequently increased [23,25]. SOS induction also plays an important role in persister survival during the post-antibiotic recovery phase after fluoroquinolone treatments [26,27,28].

Toxin-antitoxin (TA) systems are implicated in several processes, including stress adaptation, genomic stabilization, and phage abortive infection [29,30,31]. They are classified into different types according to the mechanism by which the antitoxin controls its cognate toxin [32]. TA systems are also suspected to halt cell growth and directly favor persister formation under stress conditions. Indeed, the SOS-induced toxin gene *tisB* from the type I TA system *tisB*/*istR-1* was shown to influence persistence upon DNA damage in *E. coli* [25,33,34]. The LexA-box in the promoter region of *tisB* has a very low HI and transcription is, therefore, strongly induced under DNA-damaging conditions [35]. However, translation is tightly regulated by the 5′ untranslated region (UTR) of *tisB* mRNA and RNA antitoxin IstR-1. The primary *tisB* transcript (+1 mRNA) is translationally inactive due to a 5′ UTR structure, which sequesters a so-called ribosome standby site (RSS) and thus prevents pre-loading of the 30S ribosomal subunit [36]. Upon processing of the primary transcript to the +42 mRNA, the RSS becomes accessible for 30S binding, which is promoted by a pseudoknot at the +42 mRNA 5′ end and ribosomal protein S1 [37,38]. However, under non-stress conditions, translation is counteracted by binding of antitoxin IstR-1 to the RSS. Upon IstR-1 binding, the RNA duplex is cleaved by RNase III, and the resulting +106 mRNA is translationally inactive [36,39]. The two regulatory RNA elements (5′ UTR structure in *tisB* +1 mRNA and antitoxin IstR-1) clearly restrict TisB expression to stress conditions [25,40,41,42]. TisB is a small hydrophobic protein with a length of 29 amino acids. It is located in the cytoplasmic membrane and causes the breakdown of the proton motive force, which leads to membrane depolarization and ATP depletion [25,43,44]. Since ATP depletion favors persister formation [45,46], depolarization and subsequent ATP depletion tentatively explain TisB-dependent persistence upon ciprofloxacin (Cip) treatment [25,33].

In *E. coli* wild-type cultures, TisB-dependent depolarization was only observed after prolonged Cip treatment. However, deletion of the two regulatory RNA elements (mutant Δ1-41 Δ*istR*) increased the likelihood of depolarization, even when cultures were treated with low doses of Cip [25]. Furthermore, the Δ1-41 Δ*istR* mutant has a ‘high persistence’ (*hip*) phenotype for different antibiotics during the exponential phase [25,47,48]. We have recently observed that TisB expression is uncoupled from SOS induction in the Δ1-41 Δ*istR* mutant and that the *hip* phenotype originates from LexA-independent TisB expression during the late stationary phase [42]. Here, we show that elevated TisB levels impede the expression of SOS genes, likely due to global inhibition of cellular processes. Treatment with Cip is tolerated by preventing strong DNA damage. By contrast, treatment with the DNA cross-linker mitomycin C (MMC) efficiently eradicates persisters in Δ1-41 Δ*istR* cultures. Our study demonstrates that the *hip* phenotype is conditional, and that post-transcriptional regulation of *tisB* likely ensures maximal fitness under a variety of environmental conditions.

## 2. Materials and Methods

### 2.1. Growth Conditions

All strains used in this study were derived from *E. coli* K-12 wild type MG1655 (Table S1) and grown in lysogeny broth (LB) at 37 °C with orbital shaking (180 rpm). If applicable, selection makers were added at the following concentrations: 200 µg mL^−1^ ampicillin, 50 µg mL^−1^ kanamycin, 15 µg mL^−1^ chloramphenicol, and 6 µg mL^−1^ tetracycline. Inoculation was performed by transferring a single colony into a fresh medium and incubating overnight. Pre-cultures were diluted 100-fold into fresh LB medium. Optical density measurements at 600 nm (OD_600_) were applied to monitor growth using a Cell density meter model 40 (Fisher Scientific, Schwerte, Germany).

### 2.2. Plasmid and Strain Construction

For the construction of pBAD-*syfp2*, the *syfp2* gene was PCR-amplified using primers syfp2-for-Eco and syfp2-rev-Hind. A modified pBAD vector [43] was amplified with primers topo-fw-Hind and topo-rev-Eco. PCR products were digested with EcoRI and HindIII (FastDigest; Thermo Fisher Scientific, Schwerte, Germany) and ligated using T4 DNA ligase (New England Biolabs, Ipswich, MA, USA). The final construct was confirmed by sequencing (Microsynth SeqLab, Göttingen, Germany). Chromosomal deletions and *syfp2* fusions were constructed using the heat-inducible λ red system as described in detail elsewhere [25,47,49,50]. If applicable, chromosomal deletions were moved to recipient strains using P1 transduction. Target-specific screening PCRs were performed to confirm chromosomal constructs. All primers for plasmid and strain construction are listed in Table S2.

### 2.3. Microplate Reader Experiments

Reporter strains containing chromosomal *syfp2* constructs were grown in LB medium and treated with Cip (0.1 µg mL^−1^; 10× MIC) or MMC (4× MIC; MG1655: 2.5 µg mL^−1^; Δ1-41 Δ*istR*: 10 µg mL^−1^) during the exponential phase (OD_600_ of ~0.4). sYFP2 expression from plasmid pBAD-*syfp2* was induced with 0.2% L-arabinose (L-ara) at indicated time points. For recovery experiments, Cip was removed by washing with 0.9% NaCl, and dilution of cells into a fresh medium. Cell numbers were adjusted to corresponding persister levels. Incubation was performed with continuous shaking at 37 °C in an Infinite M Nano^+^ microplate reader (Tecan, Männedorf, Switzerland) using transparent 96-well plates (Greiner Bio-One, Frickenhausen, Germany). sYFP2 fluorescence was monitored with excitation and emission wavelengths of 510 and 540 nm, respectively. The gain was set to 90 for chromosomal *syfp2* constructs, and to 50 for pBAD-*syfp2*. Optical density was measured at 600 nm. For chromosomal *syfp2* reporter strains, fluorescence values were background-corrected (LB auto-fluorescence) and normalized to OD_600_. Fluorescence values from pBAD-*syfp2* expression experiments were normalized to OD_600_.

### 2.4. Microscopy

Microscopy experiments were performed with a Leica DMI 6000 B inverse microscope (Leica Camera AG, Wetzlar, Germany) using an HCX PL APO 100×/1.4 differential interference contrast (DIC) objective. Images were recorded with a pco.edge sCMOS camera (PCO AG, Kelheim, Germany). For fluorescence images, a custom filter set (T495lpxr, ET525/50m; Chroma Technology, Bellows Falls, VT, USA) was applied. The VisiView software (Visitron Systems GmbH, Puchheim, Germany) was used for image recording and images were processed with the ImageJ-based Fiji tool (version 1.52p).

### 2.5. Flow Cytometry

Cell samples were withdrawn during the exponential phase (OD_600_ of ~0.4) and at indicated time points, washed with 1× PBS, fixed with paraformaldehyde (4% in 1× PBS) for 30 min on ice, and stored at 4 °C until measurements. Flow cytometry experiments were performed with a FACSCalibur (BD Bioscience, San Jose, CA, USA) using the CellQuest Pro 4.0.2 (BD) software. Samples were acquired using the forward scatter (Amp: 10^2^, Amp gain: 1.00), side scatter (500 V, Amp gain: 1.00) to exclude debris and a fluorescence detector FL1-H (excitation: 488 nm, emission: 530 nm, 500 V, Amp gain: 1.00) for the relative quantification of sYFP2 signals. Analysis was performed with normalized data sets (DownSample 2.0.0 plugin; 10,000 events) using FlowJo v. 10.6.2 (BD). R package *ggplot2* (version 3.3.2) with function *geom_density* and count variables was used to draw smoothed distribution plots.

### 2.6. Persister Assays

Pre-cultures for persister assays were incubated for 20 h in LB medium. If applicable, appropriate selection markers were added. Experimental cultures were prepared by 100-fold dilutions of overnight cultures into fresh LB medium without additives and incubated until the exponential phase was reached (OD_600_ of ~0.4). Treatments were performed with Cip at a final concentration of 1 µg mL^−1^ (100× MIC), or MMC at final concentrations of 10 µg mL^−1^ (4× MIC) for MG1655 or 2.5 µg mL^−1^ (4× MIC) for Δ1-41 Δ*istR* for six hours. Pre- and post-treatment samples were withdrawn and serial dilutions (in 0.9% NaCl) were plated on LB agar plates supplemented with 20 mM MgSO_4_. Colony counts were determined after 24 h and 40 h for pre- and post-treatment samples, respectively, in order to calculate persister levels.

### 2.7. ATP Measurements

ATP levels were determined as previously described [42] using the BacTiter-Glo Microbial Cell Viability Assay (Promega, Madison, WI, USA). Relative light units (RLU) were background-corrected (plain LB medium) and normalized to OD_600_.

### 2.8. DNA Damage Assay

Strains harboring pBAD-*syfp2* plasmids were grown to the exponential phase (OD_600_ of ~0.4) and treated with Cip (0.1 µg mL^−1^; 10× MIC) for two hours. Plasmid DNA from 20 mL culture was extracted using the NucleoSpin Plasmid purification kit (Macherey-Nagel, Düren, Germany) according to the manufacturer’s instructions. Then, 200 ng DNA were linearized by HindIII (FastDigest; Thermo Fisher Scientific, Schwerte, Germany) digestion at 37 °C for four hours and separated on 0.7% agarose gels containing 1× TBE. GeneRuler 1 kb Plus DNA ladder (Thermo Fisher Scientific, Schwerte, Germany) was used as a size marker. DNA was detected by ethidium bromide staining.

### 2.9. RNA Methods

The hot acid-phenol method was applied to isolate total RNA as described [35]. The quality of ribosomal RNA was assessed on 1% agarose gels, containing 1× TBE and 25 mM guanidinium thiocyanate, followed by staining with ethidium bromide. For quantitative RT-PCR (qRT-PCR), DNA-free RNA was isolated using the NucleoSpin RNA kit according to the manufacturer’s protocol (Macherey-Nagel, Düren, Germany). The Brilliant III Ultra-Fast SYBR Green QRT-PCR Master Mix (Agilent Technologies, Santa Clara, CA, USA) was applied using DNA-free RNA in a final concentration of 1 ng µL^−1^. The CFX Connect Real-Time System (Bio-Rad, Hercules, CA, USA) and the CFX Maestro Software (Bio-Rad) were used to determine cycle threshold (Ct) values. Relative transcript levels [51] were determined using either *hcaT* (exponential-phase samples) or *cysG* (Cip-treated samples) as reference for normalization [35,52]. Primers for qRT-PCR can be found in Table S2.

### 2.10. Western Blot Analysis

For immunodetection of 3×FLAG-TisB, cell samples were harvested by centrifugation (11,000× *g*, 3 min) and resuspended in SDS sample buffer. Samples were incubated at 95 °C for 5 min and subsequently subjected to Tricine-SDS-PAGE followed by semi-dry electroblotting [53]. Proteins were blotted onto PVDF membranes (Immobilon-P, 0.45 µm; Merck, Darmstadt, Germany). 3×FLAG-tagged proteins were detected using a monoclonal ANTI-FLAG M2-Peroxidase (HRP) antibody (Merck, Darmstadt, Germany). Further details were described previously [42].

### 2.11. Statistical Analysis

Statistical analysis was performed using R statistical language (Version 3.6.0; https://www.r-project.org/; date accessed: 5 July 2019). Two-tailed Welch’s *t*-test was performed on log_10_-transformed data. Normality was assessed using the Shapiro–Wilk test. *p*-value adjustment was performed by pairwise comparison according to the Holm–Bonferroni method (*p*-values < 0.05 were considered as significant). For statistical analysis of flow cytometry data, Van der Waerden test with a post-hoc pairwise comparison was applied using package PMCMR (*p*-values < 0.001 were considered as significant).

## 3. Results

### 3.1. Persisters in Mutant Δ1-41 ΔistR Neither Experience Strong DNA Damage nor Rely on Double-Strand Break Repair upon Ciprofloxacin Treatment

**Figure 1:**
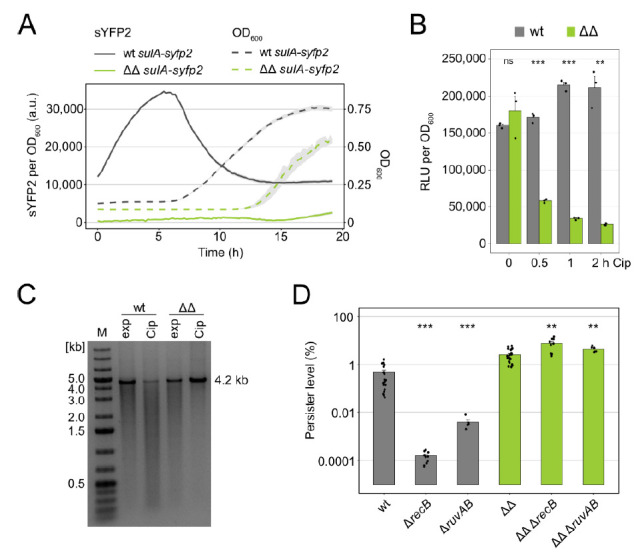
DSB repair is not crucial for persisters of mutant Δ1-41 Δ*istR*. (**A**) SOS induction during recovery. Strains were treated with Cip (0.1 µg mL^−1^; 10× MIC) for two hours during the exponential phase (OD_600_ of ~0.4). Cip was removed by washing, cells were resuspended in a fresh medium and transferred to 96-well plates. Growth (dotted lines) and sYFP2 fluorescence from chromosomal *sulA-syfp2* fusions (solid lines) were monitored in a microplate reader. Fluorescence measurements were normalized to the corresponding OD_600_ measurements. Data represent the mean (colored lines) and SEM (grey ribbon; *n* = 3). (**B**) ATP levels were determined by a luciferase-based assay upon treatment with Cip (0.1 µg mL^−1^; 10× MIC). Relative light units (RLU) were background-corrected and normalized to OD_600_. Bars represent the mean (± SEM; *n* = 3). A pairwise *t*-test was performed to compare wt and ΔΔ at each time point (ns: not significant, ** *p* < 0.01, *** *p* < 0.001). Black dots indicate the results of individual biological experiments. (**C**) Linearized plasmid DNA was analyzed on an agarose gel. Plasmid DNA was extracted from cultures in exponential phase (exp, OD_600_ of ~0.4) and after two hours of Cip treatment (0.1 µg mL^−1^; 10× MIC). A marker (M) in kb is shown on the left-hand side. (**D**) Colony counts were determined before and after six hours of Cip treatment (1 µg mL^−1^; 100× MIC) to calculate persister levels. Bars represent the mean (± SEM; *n* ≥ 6). A pairwise *t*-test was performed to compare *recB* and *ruvAB* deletions to their parental strains (** *p* < 0.01, *** *p* < 0.001). (wt: wild type MG1655; ΔΔ: Δ1-41 Δ*istR*). Black dots indicate the results of individual biological experiments.

We have recently observed that persister cells in exponential-phase cultures of mutant Δ1-41 Δ*istR* (from now on ΔΔ) are carried over from stationary phase, where they are formed due to SOS-independent TisB expression. Hence, the *hip* phenotype of this mutant does not depend on SOS induction through LexA degradation, as shown by experiments with the non-cleavable LexA variant LexA3 [42]. However, it was not addressed whether ΔΔ persisters experience DNA damage or rely on DNA repair mechanisms. Since the *hip* phenotype of mutant ΔΔ is best documented in the exponential phase [25,42,47,48], we performed Cip treatments when an optical density at 600 nm (OD_600_) of ~0.4 was reached. Mutant ΔΔ was compared to its parental strain *E. coli* K-12 wild type MG1655. From *E. coli* wild-type persisters it is known that activation of the SOS response and repair of DNA damage is especially important for survival during the early recovery phase after fluoroquinolone treatments [26,27,28]. Therefore, we tested the activation of the SOS response in ΔΔ cultures during post-antibiotic recovery by measuring a transcriptional *sulA-syfp2* reporter fusion, which is a valuable read-out for SOS induction [26,28]. Cells were treated with Cip for two hours, washed, diluted into a fresh medium, and transferred to microtiter plates to measure growth (OD_600_) and sYFP2 fluorescence over time. Immediately after the transfer, wild-type cultures scored high fluorescence values of ~12,000 arbitrary units (a.u.). By contrast, fluorescence values in ΔΔ cultures were quite low (<500 a.u.; Figure 1A). This considerable difference was due to high *sulA-syfp2* expression in wild-type cultures after two hours of Cip treatment (Figure S1). Shortly after the transfer, wild-type cultures exhibited a further increase in sYFP2 fluorescence, reaching ~34,000 a.u. after six hours of recovery. Maximum sYFP2 fluorescence preceded growth resumption by one hour, as judged from an increase in OD_600_ at around seven hours recovery (Figure 1A). Upon growth resumption, sYFP2 fluorescence steadily declined over time. These findings were consistent with single-cell observations of *E. coli* wild-type persisters treated with the fluoroquinolone ofloxacin during the exponential phase [28]. The ΔΔ mutant showed a very different pattern. First, growth resumption after Cip treatment was clearly shifted to a later time point (from ~7 to ~13 h recovery; Figure 1A). We have recently observed that the lag phase after dilution of stationary-phase cultures was extended by ~60 min in mutant ΔΔ compared to wild type [42]. The six-hour shift in growth resumption, as observed here (Figure 1A), clearly confirms the delayed post-antibiotic recovery of ΔΔ cells [25,47,48]. Second, sYFP2 fluorescence stayed at a low level over the whole recovery period (Figure 1A). Hence, the ΔΔ mutant did not induce the SOS response during recovery.

Our observations raised the question of whether ΔΔ persisters experienced strong DNA damage at all. TisB expression causes ATP depletion [43], and ATP depletion itself was shown to avoid Cip-induced DSBs [45]. We first assessed ATP levels by a luciferase-based assay. In wild-type cultures, ATP levels stayed stable or were even slightly increased (1.3-fold), during the first two hours of Cip treatment (Figure 1B). In ΔΔ cultures, ATP levels dropped by ~3-fold already after 30 min and by ~7-fold after two hours (Figure 1B). These findings support the prevailing model that TisB-dependent depolarization leads to ATP depletion [25,33,43,44]. To assess the occurrence of DSBs, plasmid DNA was extracted from wild-type and ΔΔ cells before and after two hours of Cip treatment. The integrity of linearized plasmids was analyzed on agarose gels. Wild-type cells had a clear reduction in full-length plasmids and an increased fraction of shorter fragments, indicative of Cip-induced DSBs (Figure 1C). By contrast, ΔΔ cells mainly contained full-length plasmids (Figure 1C), suggesting that ΔΔ cells did not experience strong DNA damage. However, a time-course experiment indicated that minor DNA damage might occur in ΔΔ cells at least during the first 30 min of Cip treatment (Figure S2).

Together, the above data suggested that ΔΔ persisters do not rely on DNA repair in order to survive a Cip treatment. To test this possibility, persister assays were performed with *recB* and *ruvAB* deletion strains. Both the RecBCD enzyme complex and the RuvAB Holliday junction complex are important components of DSB repair via homologous recombination. Deletion of *recB* and *ruvAB* in the wild-type background reduced persister levels upon Cip treatment by more than 3000-fold and 100-fold, respectively (Figure 1D). Similar results were already obtained in other studies [23,24]. By contrast, in the ΔΔ background, persister levels even slightly increased due to the *recB* and *ruvAB* deletions (Figure 1D). These experiments demonstrated that ΔΔ persisters do not rely on DSB repair. We note that the persister level of ~3% in ΔΔ cultures (Figure 1D) does not reflect the population-wide protection against DNA damage (Figure 1C). We speculate that most ΔΔ cells die due to Cip-induced TisB overexpression (see Discussion) but cannot exclude the involvement of other factors.

### 3.2. TisB Overexpression in Mutant Δ1-41 ΔistR upon Ciprofloxacin Treatment

**Figure 2:**
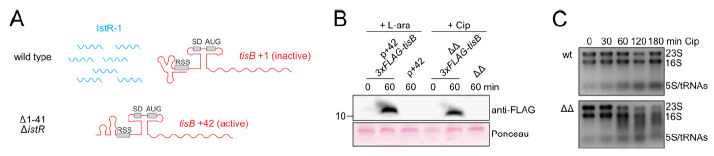
TisB overexpression in mutant Δ1-41 Δ*istR*. (**A**) Graphical illustration of RNA species expressed from the *tisB/istR-1* system. In the wild type, RNA antitoxin IstR-1 and the translationally inactive *tisB* +1 mRNA are present. In mutant ΔΔ, transcription from the LexA-dependent promoter produces the translationally active *tisB* +42 mRNA. (**B**) Western blot analysis of 3×FLAG-TisB, either expressed from plasmid p+42-*3*×*FLAG-tisB* by addition of L-ara (0.2%), or from the ΔΔ-*3*×*FLAG-tisB* locus by addition of Cip (0.1 µg mL^−1^; 10× MIC) for 60 min. Total protein was separated by Tricine-SDS-PAGE. 3×FLAG-TisB was detected using an anti-FLAG antibody. Constructs without *3*×*FLAG* sequence served as specificity controls. Western membranes were stained with Ponceau as a loading control. (**C**) Total RNA was isolated during exponential phase (OD_600_ of ~0.4) and at different time points during Cip treatment (0.1 µg mL^−1^; 10× MIC). RNA quality was analyzed on agarose gels. rRNAs (23S, 16S, and 5S) and tRNAs are indicated. (wt: wild type MG1655; ΔΔ: Δ1-41 Δ*istR*).

The *tisB* promoter is very sensitive to DNA damage [21,35], and even minor DNA damage, as observed early during a Cip treatment (Figure S2), is expected to cause *tisB* transcription in ΔΔ cells. Indeed, increased *tisB* +42 mRNA levels were observed in the ΔΔ mutant upon Cip treatment [25]. Due to the lack of post-transcriptional *tisB* repression in mutant ΔΔ (Figure 2A), increased TisB protein levels can be expected as well. Chromosomal insertion of a *3*×*FLAG* sequence into the *tisB* gene (between codon 2 and 3) allowed us to detect 3×FLAG-TisB expressed from the ΔΔ locus upon treatment with the DNA-damaging antibiotic Cip. Importantly, the N-terminal 3×FLAG-tag does not affect TisB localization or functionality [43]. Western blot analysis revealed that expression of 3×FLAG-TisB from the ΔΔ locus was comparable to induction of 3×FLAG-TisB from plasmid p+42-*3*×*FLAG-tisB* [43] using L-arabinose (L-ara) as an inducer (Figure 2B). Hence, Cip treatment caused overexpression of TisB in the ΔΔ mutant. Since ectopic overexpression of TisB causes rRNA degradation [43,47], we isolated total RNA from wild-type and ΔΔ cultures. Progressive rRNA degradation was observed in ΔΔ cultures after 60 min of Cip treatment, while rRNA remained intact for 180 min in wild-type cultures (Figure 2C). These data demonstrated that ΔΔ cells were clearly affected by elevated levels of the membrane-targeting toxin TisB.

### 3.3. Major Cellular Processes Are Inhibited in Mutant Δ1-41 ΔistR upon Ciprofloxacin Treatment

**Figure 3:**
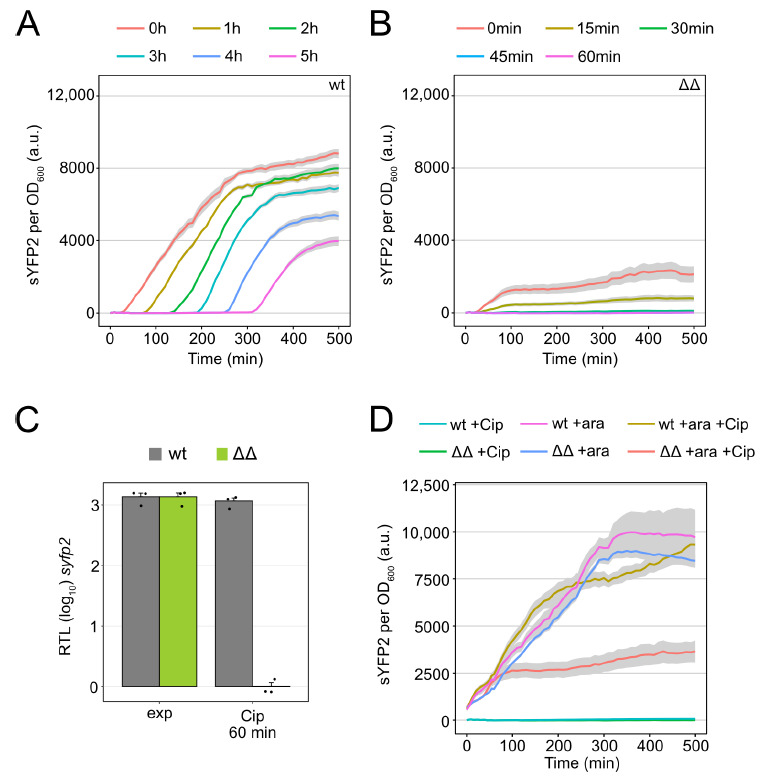
Gene expression is inhibited in mutant Δ1-41 Δ*istR* upon ciprofloxacin. (**A**,**B**) sYFP2 fluorescence from plasmid pBAD-*syfp2* was monitored in a microplate reader. Strains wt pBAD-*syfp2* (**A**) and ΔΔ pBAD-*syfp2* (**B**) were treated with Cip (0.1 µg mL^−1^; 10× MIC) during the exponential phase (OD_600_ of ~0.4), which corresponds to time point 0 min. sYFP2 expression was induced by adding 0.2% L-Ara at the indicated time points during Cip treatment. Fluorescence measurements were normalized to the corresponding OD_600_ measurements. Data represent the mean (colored lines) and SEM (grey ribbons; *n* = 3). (**C**) Relative transcript levels (RTL, log_10_) were calculated by qRT-PCR to assess *syfp2* induction upon treatment with L-ara. RNA samples were collected before and 30 min after induction with 0.2% L-ara, either during the exponential phase (exp, OD_600_ of ~0.4) or after 60 min of Cip treatment (0.1 µg mL^−1^; 10× MIC). Bars represent the mean (± SEM; *n* = 3). Black dots indicate the results of individual biological experiments. (**D**) sYFP2 fluorescence from plasmid pBAD-*syfp2* was monitored in a microplate reader. sYFP2 expression was induced by 0.2% L-Ara (+ara) 30 min before treatment with Cip (0.1 µg mL^−1^; 10× MIC). Treatments with L-ara or Cip alone served as controls. Fluorescence measurements were normalized to the corresponding OD_600_ measurements. Data represent the mean (colored lines) and SEM (grey ribbons; *n* = 3). (wt: wild type MG1655; ΔΔ: Δ1-41 Δ*istR*).

It has been shown that ectopic overexpression of TisB causes rapid shutdown of major cellular processes, including transcription and translation [43], likely affecting global gene expression. Here, induction of the *syfp2* gene from plasmid pBAD-*syfp2* was monitored to evaluate gene expression in the ΔΔ mutant upon Cip treatment. Cells were exposed to Cip and subsequently treated with L-ara to induce the *syfp2* gene at different time points during the Cip treatment. In wild-type cultures, the addition of L-ara caused an immediate increase in sYFP2 fluorescence, even after five hours of Cip treatment (Figure 3A). By contrast, when L-ara was added to ΔΔ cultures at the beginning of the Cip treatment, the increase in sYFP2 fluorescence was clearly diminished (Figure 3B). More intriguingly, an increase in sYFP2 fluorescence was absent in ΔΔ cultures when L-ara was added as early as 30 min after the onset of Cip treatment (Figure 3B).

To test whether this complete shutdown of gene expression already occurred on the level of transcription, mRNA levels of *syfp2* were monitored using quantitative RT-PCR. Under non-stress conditions, *syfp2* mRNA levels were strongly induced (~1370-fold) by L-ara in both wild-type and ΔΔ cultures (Figure 3C). By contrast, 60 min after Cip treatment, *syfp2* mRNA levels were not inducible at all in ΔΔ cultures, while wild-type cultures still showed strong induction (~1160-fold). We conclude that TisB overexpression either completely shuts down transcription or interferes with the uptake of L-ara, which seems plausible since uptake systems depend on either the proton gradient (AraE) or ATP (AraFGH), both of which are exhausted by the action of TisB [25,43,44]. In a control experiment, cultures were pre-treated with L-ara for 30 min, to enable maximum uptake of the inducer, and only subsequently exposed to Cip. Wild-type cultures exhibited a steadily increasing sYFP2 fluorescence. In ΔΔ cultures, sYFP2 signals increased for ~75 min at a rate that was comparable to the wild type. Afterward, the increase in sYFP2 fluorescence was clearly reduced, but not completely abolished (Figure 3D). When ΔΔ cells were treated with L-ara alone, sYFP2 fluorescence steadily increased over time, reaching maximum levels comparable to wild-type experiments (Figure 3D). Treatment with Cip alone did not cause an increase in sYFP2 fluorescence, demonstrating that Cip itself had no influence on the reporter (Figure 3D). In summary, we conclude that TisB overexpression in ΔΔ cultures causes the shutdown of energy-dependent transport processes due to membrane depolarization and ATP depletion, which completely hinders L-ara uptake already after ~30 min of Cip treatment. Upon ongoing TisB overexpression (after ~75 min), gene expression is negatively affected, likely due to a shortage of ATP.

Conclusions drawn from toxin overexpression experiments might not directly apply to the wild-type situation. In order to show that the negative influence of TisB on gene expression also occurs in the wild type, a *tisB* deletion strain was investigated. Measurements with the inducible *syfp2* reporter system (plasmid pBAD-*syfp2*) demonstrated that the *tisB* deletion strain scored higher sYFP2 fluorescence values than the wild type, which was particularly evident after prolonged treatment with Cip (Figure S3). These data suggest that, in wild-type cells, increasing TisB amounts inhibit gene expression upon extended periods of DNA damage.

### 3.4. High TisB Levels Counteract Expression of SOS Genes

**Figure 4:**
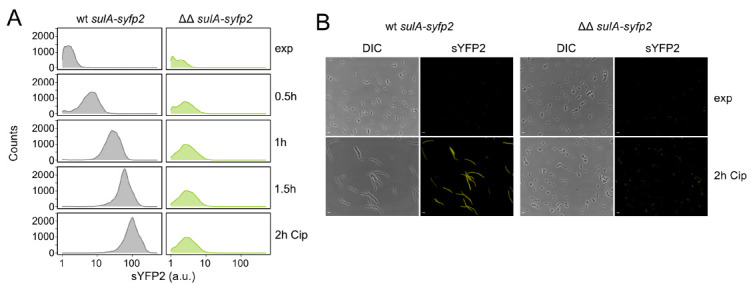
Inhibition of *sulA* expression in mutant Δ1-41 Δ*istR* upon ciprofloxacin. (**A**) Flow cytometry analysis of *sulA-syfp2* fusion strains treated with Cip. Cell samples were taken during the exponential phase (exp, OD_600_ of ~0.4) and at indicated time points of Cip treatment (0.1 µg mL^−1^; 10× MIC). Event counts were normalized to 10,000 events. (**B**) Differential interference contrast (DIC) and fluorescence (sYFP2) microscopy images of strains harboring chromosomal *sulA-syfp2* fusions during exponential phase (exp, OD_600_ of ~0.4) and after two hours of Cip treatment (0.1 µg mL^−1^; 10× MIC). Scale bars indicate 5 µm. (wt: wild type MG1655; ΔΔ: Δ1-41 Δ*istR*).

We have so far shown that the ΔΔ mutant strongly produces TisB upon Cip treatment (Figure 2B), likely due to minor DNA damage (Figure S2), and that strong TisB production is linked to an overall reduction in gene expression (Figure 3). It remains, however, unknown to which extent other SOS genes are induced in the ΔΔ mutant. In order to assess Cip-dependent SOS induction, the transcriptional *sulA-syfp2* reporter fusion was applied. A steady, population-wide increase in sYFP2 fluorescence from the *sulA* locus was detected only in wild-type cultures, as revealed by flow cytometry (Figure 4A). After two hours of Cip treatment, the median sYFP2 fluorescence had increased significantly (~61-fold). These findings were supported by sYFP2 measurements with another SOS reporter construct (*dinB-syfp2*; Figure S1). The ΔΔ mutant only exhibited a very slight, albeit significant, increase in sYFP2 fluorescence of ~2-fold during the first hour of Cip treatment, but median fluorescence values stayed stable afterward (Figure 4A). We note that the one-hour time frame of sYFP2 production from the *sulA-syfp2* reporter in ΔΔ cells perfectly matches our observations with the inducible *syfp2* system (Figure 3). Furthermore, fluorescence microscopy revealed that Cip-induced and SulA-dependent cell filamentation [54,55] was absent in ΔΔ cultures (Figure 4B). The filamentation phenotype was restored by ectopic overexpression of antitoxin IstR-1 in ΔΔ (Figure S4), suggesting that lack of filamentation in ΔΔ cultures was due to a TisB-dependent defect in SulA expression.

### 3.5. The Hip Phenotype of Mutant Δ1-41 ΔistR Is Lost upon Treatment with the DNA Cross-Linker Mitomycin C

**Figure 5:**
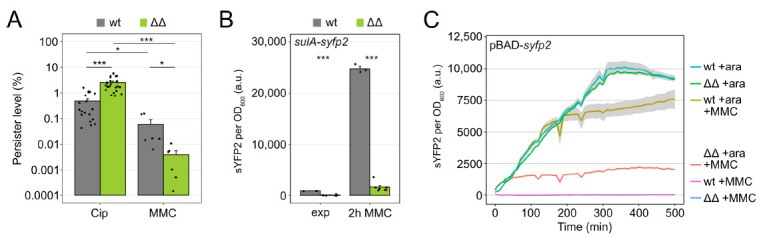
Mutant Δ1-41 Δ*istR* is highly susceptible to mitomycin C. (**A**) Colony counts were determined before and after six hours of Cip (1 µg mL^−1^; 100× MIC) or MMC treatment (wt: 10 µg mL^−1^; ΔΔ: 2.5 µg mL^−1^; 4× MIC). Colony counts were used to calculate persister levels. Bars represent the mean (± SEM; *n* ≥ 6). A pairwise *t*-test was performed to compare persister levels (* *p* < 0.05, *** *p* < 0.001). Black dots indicate the results of individual biological experiments. (**B**) sYFP2 fluorescence from a chromosomal *sulA-syfp2* fusion was monitored in a microplate reader. Reporter strains were analyzed during the exponential phase (exp, OD_600_ of ~0.4) and after two hours of MMC treatment (wt: 10 µg mL^−1^; ΔΔ: 2.5 µg mL^−1^; 4× MIC). Fluorescence measurements were background-corrected and normalized to OD_600_. Bars represent the mean (± SEM; *n* ≥ 3). A pairwise *t*-test was performed to compare wt and ΔΔ (*** *p* < 0.001). Black dots indicate the results of individual biological experiments. (**C**) sYFP2 fluorescence from plasmid pBAD-*syfp2* was monitored in a microplate reader. sYFP2 expression was induced by 0.2% L-Ara (+ara) 30 min before treatment with MMC (wt: 10 µg mL^−1^; ΔΔ: 2.5 µg mL^−1^; 4× MIC). Treatments with L-ara or MMC alone served as controls. Fluorescence measurements were normalized to the corresponding OD_600_ measurements. Data represent the mean (colored lines) and SEM (grey ribbons; *n* = 3). (wt: wild type MG1655; ΔΔ: Δ1-41 Δ*istR*).

Tolerance to Cip in ΔΔ persisters does not depend on DSB repair due to the prevention of strong DNA damage (Figure 1). We were curious whether the *hip* phenotype of the ΔΔ mutant would still occur when cells were treated with MMC. MMC is a potent DNA cross-linker, which is effective against persisters from different bacterial species [56]. The MIC for MMC was four-fold higher in the wild type compared to the ΔΔ mutant (2.5 µg/mL versus 0.625 µg/mL, respectively; Figure S5). For persister assays, the MMC concentration was adjusted to 4× MIC (10 µg/mL for wild type and 2.5 µg/mL for ΔΔ). In both strains, MMC was more effective against persisters than Cip (Figure 5A). However, in ΔΔ cultures, the persister level was ~15-fold lower than in wild-type cultures (0.004% versus 0.06%, respectively; Figure 5A), demonstrating that the ΔΔ mutant was highly susceptible to MMC. Similar to what was observed upon Cip treatment, SOS induction (as measured by the *sulA-syfp2* reporter fusion) was largely suppressed in the ΔΔ mutant upon MMC treatment (Figure 5B), and induction was also not observed in the post-antibiotic recovery phase (data not shown). Furthermore, inhibition of gene expression (as measured by pBAD-*syfp2*) occurred in MMC-treated ΔΔ cells (Figure 5C). These data suggest that ΔΔ persisters fail to survive an MMC treatment due to their inability to induce the SOS response. In this particular case, and in contrast to Cip (Figure 5A), ΔΔ cells are even more likely to perish than wild-type cells.

## 4. Discussion

Persister cells are marked by their ability to tolerate high levels of antibiotics and resume growth after the antibiotic treatment has ceased. While dormancy is generally expected to favor persistence, it is not necessary for persistence to occur. Persister cells might retain metabolic activity or even actively extrude antibiotics [12,14,15]. However, inactivation of distinct cellular processes clearly supports persister formation, as exemplified by ribosome hibernation [57], reduced uptake of antibiotics [58], treatment with bacteriostatic agents [59], and expression of toxins from TA systems [60,61]. The small membrane-targeting toxins TisB, HokB, and GhoT have been implicated in bacterial persistence due to their ability to reduce the proton motive force and deplete cellular ATP levels [12,25,33,62,63,64]. While GhoT belongs to a type V TA system [63], TisB and HokB are toxins from type I TA systems. A hallmark of type I TA systems is tight regulation of toxin expression at the post-transcriptional level. Primary transcripts of toxin genes are translationally inert due to intrinsic secondary structures that prevent ribosome binding and translation initiation, which leads to transcription-translation uncoupling. Activation of primary transcripts involves a processing step that enables structural rearrangements and ribosome accessibility [36,65,66,67,68]. However, processed mRNAs are bound by cognate RNA antitoxins, which triggers degradation by RNase III. We refer the reader to recent reviews for more mechanistic details on post-transcriptional regulation in type I TA systems [41,69,70,71,72]. It is intuitive to assume that such sophisticated regulation serves a purpose, such as avoidance of toxin overexpression and concomitant side effects, some of which were revealed in the current study.

Past experiments have demonstrated that mutant ΔΔ has a *hip* phenotype upon treatment with fluoroquinolones and β-lactams [25,47,48]. It was assumed that the regulatory mutant rapidly produces TisB upon DNA damage, which allows many cells to enter the persister state before detrimental DNA damage occurs [25]. However, we only recently observed that the regulatory mutant produces TisB during the late stationary phase in an SOS- and LexA-independent manner, and that stationary-phase expression of TisB gives rise to a subpopulation of growth-retarded cells that are likely to be scored as persisters upon Cip treatment [42]. Here, we show that mutant ΔΔ has strongly elevated TisB levels during the exponential phase upon Cip treatment (Figure 2B). We assume that all actively growing cells will quickly overproduce TisB in an SOS- and LexA-dependent manner, while the pre-existing and growth-retarded subpopulation is not prone to further TisB production. Even though all cells are expected to experience ATP depletion (Figure 1B), thereby preventing strong Cip-induced DNA damage (Figure 1C) [45], only the pre-existing subpopulation contributes to the *hip* phenotype [42]. Since ectopic overexpression of TisB reduces viable cell counts by at least 10-fold [43], it seems likely that the actively growing part of the population is not killed by Cip-induced DNA damage but rather Cip-induced TisB overexpression.

It appears that cell death occurs in mutant ΔΔ due to strong TisB expression upon Cip treatment. It is known that ectopic overexpression of TisB causes rRNA degradation and shutdown of major cellular processes [43,47]. Here, the same was observed in the regulatory mutant after ~60 min of Cip treatment (Figure 2C and Figure 3), indicating that a threshold of TisB protein was reached, beyond which most cellular processes are strongly impeded. The rRNA degradation is indicative of ribosome destabilization, which together with enhanced ATP depletion would largely explain the shutdown of protein biosynthesis. In *Helicobacter pylori*, ectopic expression of toxin AapA1 and concomitant rRNA degradation are correlated with cell death [65]. However, a direct causal link between rRNA degradation and cell death is lacking for mutant ΔΔ. On the contrary, we have indications that rRNA degradation does not affect survival (our unpublished data). We rather suggest that rRNA degradation is an unwanted side effect in the regulatory mutant, possibly contributing to a deeper state of dormancy and an extended period of post-antibiotic recovery of TisB-dependent persister cells.

Several studies have emphasized the importance of SOS induction and DNA repair during the post-antibiotic recovery phase after fluoroquinolone treatments [26,27,28]. According to this view, the persister phenotype depends on an active mechanism that follows the antibiotic treatment. Alternatively, the persister phenotype is established due to the inactivation of antibiotic targets, for example, by strong ATP depletion [45,46]. Fluoroquinolones cause DSBs by stabilizing DNA-cleavage complexes formed by topoisomerase II (gyrase) or IV. Accumulation of DSBs is largely avoided by ATP depletion and concomitant inactivation of topoisomerases [45]. TisB-induced persistence clearly conforms to the latter model, underscoring the importance of cellular inactivation for persistence.

Interestingly, our data indicate that strong TisB production impedes the expression of SOS genes (Figure 4), thereby corrupting the induction of DNA repair systems. However, when treated with Cip, this disadvantage is not apparent because strong DNA damage is prevented (Figure 1C). Hence, SOS induction and DSB repair systems are dispensable for persister survival in the particular case of the ΔΔ mutant (Figure 1D). MMC, on the other hand, initiates crosslinking of opposing DNA strands after spontaneous reduction of the drug. Since TisB-producing cells are expected to maintain reducing power, as observed for HokB-expressing cells [12], crosslinking and DSBs cannot be avoided by the action of TisB. Now, induction of the SOS response and DNA repair systems are crucial to counteract and tolerate MMC. In this particular case, wild-type cells are better adapted, and the *hip* phenotype of mutant ΔΔ converts into a highly susceptible phenotype (Figure 5A). These experiments demonstrate that *hip* phenotypes can be conditional and that an advantage under certain conditions easily turns into a disadvantage as soon as conditions change. If persister formation is understood as a bet-hedging strategy for survival [1,62], it is certainly a benefit to bet on diverse persister types.

Finally, sYFP2 fluorescence measurements suggest that translation is inhibited upon TisB expression in mutant ΔΔ (Figure 3B,D), similar to what was observed for ectopic TisB expression [43]. Since protein synthesis is the most energy-consuming process in bacterial cells [73], TisB-dependent ATP depletion (Figure 1B) [43] is a satisfying explanation for the observed inhibition of translation. In addition, the import of sugars, such as L-ara, might be inhibited, as indicated by the lack of *syfp2* induction on the mRNA level (Figure 3C). We assume that both depolarization and ATP depletion rapidly interfere with transport over the inner membrane but cannot exclude that TisB accumulation itself has a negative influence on transport systems. Reduced uptake of sugars might also contribute to the prolonged post-antibiotic recovery time that has been observed for TisB persisters [25,47]. Recovery was even further delayed when the *ompF* gene was deleted [48]. OmpF is an outer membrane porin that provides the sugar supply to the periplasm [74]. If *ompF* is deleted in mutant ΔΔ, cells likely struggle to provide the necessary resources for initiating growth due to limited uptake at both the inner and outer membrane. Further experiments are clearly needed to evaluate the TisB-dependent effect on sugar transport and its implication for recovery.

In summary, we revealed several side effects of TisB overexpression in mutant ΔΔ, ranging from rRNA degradation to inhibition of cellular process, including expression of SOS genes and probably membrane transport. Since mutant ΔΔ is an engineered strain, it remains an important question whether similar TisB-dependent side effects also occur in wild-type cells upon DNA damage. Preliminary experiments, comparing wild type to a *tisB* deletion strain, indeed suggest that some effects also occur in wild-type cultures upon extended periods of DNA damage (Figure S3 and our unpublished data). Whether these secondary effects influence TisB-induced persistence remains an exciting question for future studies.

## Data Availability

The data presented in this study are available on request from the corresponding author.

## Supplementary Materials

The following are available online at https://www.mdpi.com/article/10.3390/microorganisms9050943/s1. Table S1: Strains and plasmids used in this study, Table S2: Oligodeoxyribonucleotides used in this study, Figure S1: Induction of SOS genes upon ciprofloxacin, Figure S2: Time course experiment for DNA damage analysis, Figure S3: sYFP2 expression upon prolonged ciprofloxacin treatment, Figure S4: Cell filamentation upon ciprofloxacin, Figure S5: MIC determination for ciprofloxacin and mitomycin C.
