# Supplementary material for: Elevated Expression of Toxin TisB Protects Persister Cells against Ciprofloxacin but Enhances Susceptibility to Mitomycin C
Source: Microorganisms. 2021 Apr 27;9(5):943. doi: 10.3390/microorganisms9050943 (PMC8145889; doi:10.3390/microorganisms9050943)
Supplement: Supplementary file 1 [file microorganisms-09-00943-s001.zip › microorganisms-1194436-SI.pdf]

Supplementary Materials

Elevated expression of toxin TisB protects persister cells against ciprofloxacin but enhances susceptibility to mitomycin C

Daniel Edelmann, Florian H. Leinberger, Nicole E. Schmid, Markus Oberpaul,

Till F. Schäberle and Bork A. Berghoff

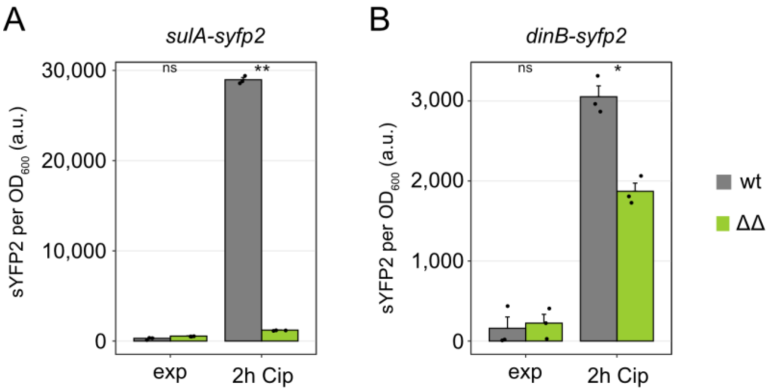

Figure S1. *Induction of SOS genes upon ciprofloxacin.*

sYFP2 fluorescence from chromosomal *sulA-syfp2* (A) and *dinB-syfp2* (B) fusions was monitored in a microplate reader. Reporter strains were analyzed during exponential phase (exp, OD_{600} of ~0.4) and after two hours of Cip treatment (0.1 µg mL^{-1}; 10x MIC). Fluorescence measurements were background-corrected and normalized to OD_{600}. Bars represent the mean (± SEM; n=3). A pairwise *t*-test was performed to compare wt and ΔΔ (ns: not significant, * $p<0.05$, ** $p<0.01$). (wt: wild type MG1655; ΔΔ: Δ1-41 Δ*istR*).

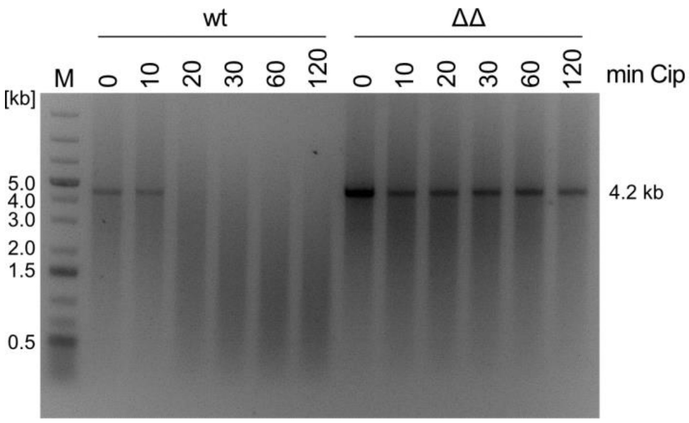

Figure S2. *Time course experiment for DNA damage analysis.*

Linearized plasmid DNA was analyzed on an agarose gel. Plasmid DNA was extracted from cultures in exponential phase (OD_{600} of ~0.4) and at several time points of Cip treatment (1 µg mL^{-1}; 100x MIC). A marker (M) in kb is shown on the left-hand side. (wt: wild type MG1655; ΔΔ: Δ1-41 Δ*istR*).

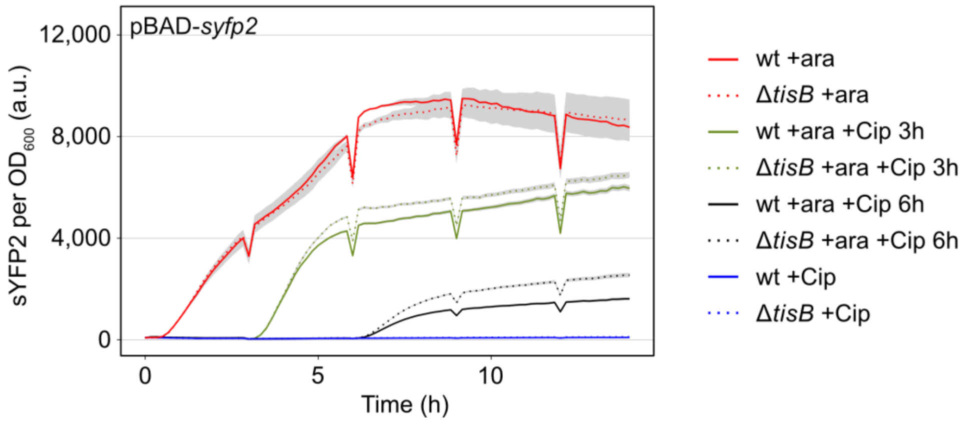

Figure S3. *sYFP2 expression upon prolonged ciprofloxacin treatment.*

sYFP2 fluorescence from plasmid pBAD-*syfp2* was monitored in a microplate reader. sYFP2 expression was induced by 0.2% L-Ara (+ara) at the indicated time points during Cip treatment (0.1 µg mL^{-1}; 10x MIC). Treatments with L-ara or Cip alone served as controls. Fluorescence measurements were normalized to the corresponding OD_{600} measurements. Data represents the mean (colored lines) and SEM (grey ribbons; n=3). (wt: wild type MG1655; Δ*tisB*: *tisB* deletion strain).

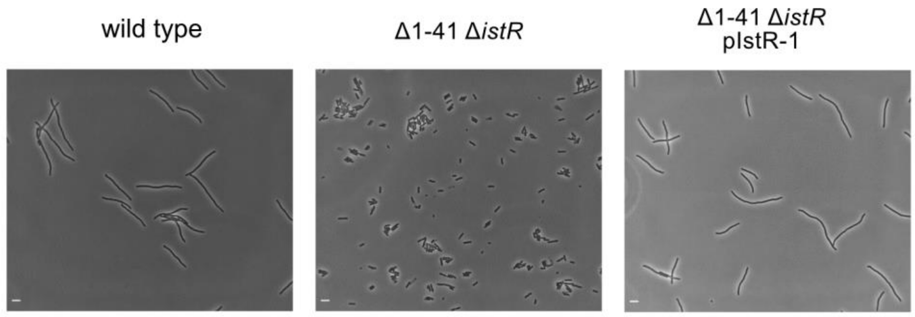

Figure S4. *Cell filamentation upon ciprofloxacin.*

Differential interference contrast (DIC) microscopy images of strains after two hours of Cip treatment (0.1 µg mL^{-1}; 10x MIC). pIstR-1 is an overexpression plasmid for RNA antitoxin IstR-1. Scale bars indicate 5 µm.

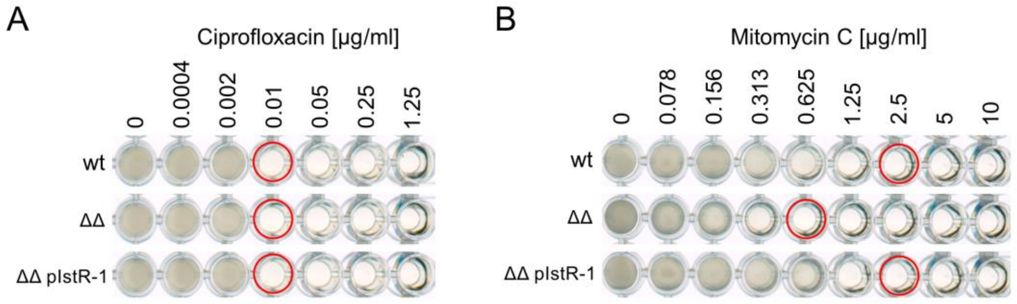

Figure S5. *MIC determination for ciprofloxacin and mitomycin C.*

Stationary-phase cultures were diluted 1:1,000 in fresh LB medium, and 135 µl were transferred to each well of a 96-well microplate. Subsequently, 15 µl of antibiotics were added to reach the final concentrations as indicated. Cells were cultivated at 37°C and 180 rpm for ~24 hours. The minimum inhibitory concentration (MIC) is reflected by the first well (red circle) in which cell growth was below 10% of the control (0 µg/ml), as assessed by OD_{600} measurements in a microplate reader. (wt: wild type MG1655; ΔΔ: Δ1-41 Δ*istR*; ΔΔ pIstR-1: Δ1-41 Δ*istR* with IstR-1 overexpression plasmid).

Table S1. Strains and plasmids used in this study.

Strain	Relevant features	Source/Reference
MG1655	K-12 F^{-} λ^{-}	Lab strain
B047	Δ*tisAB::frt* in MG1655	[1]
B133	Scarless deletion of +1-41 region of *tisB* 5'-UTR and deletion of *istR* (Δ1-41 Δ*istR::frt-kan-frt*)	[2]
B076	MG1655 *sulA-syfp2-cat*	[2]
GC-40	MG1655 *dinB-syfp2-cat*	This study
NES3	Δ1-41 Δ*istR::frt-kan-frt sulA-syfp2-cat* (P1 from B076 to B133)	This study
NES5	Δ1-41 Δ*istR::frt-kan-frt dinB-syfp2-cat* (P1 from GC-40 to B133)	This study
GC-100	Deletion of *recB* with *cat* (Δ*recB::cat*) in MG1655	This study
GC-102	Deletion of *ruvAB* with *cat* (Δ*ruvAB::cat*) in MG1655	This study
GC-103	MG1655 Δ*recB::cat* (P1 from GC-100 to MG1655)	This study
GC-106	Δ1-41 Δ*istR::frt-kan-frt* Δ*recB::cat* (P1 from GC-100 to B133)	This study
GC-105	MG1655 Δ*ruvAB::cat* (P1 from GC-102 to MG1655)	This study
DE-157	Δ1-41 Δ*istR::frt-kan-frt* Δ*ruvAB::cat* (P1 from GC-102 to B133)	This study
DE-222	Δ1-41 Δ*istR::frt-kan-frt* with *3xFLAG-tisB*	[3]

Plasmid	Relevant features	Source/Reference
pSIM5	λ red expression vector, pSC101 *ori*, $repA^{ts}$, Tet^{R}	[4]
pJV3H22	IstR-1 transcribed from P_{L} promoter in pZE12-luc, ColE1 *ori*, Amp^{R}	[5]
pBAD-*syfp2*	pBAD with *syfp2* under control of P_{BAD}, Amp^{R}	This study
pBAD+42	pBAD with *tisB* from +42 to +354, Amp^{R}	[6]
pBAD3xFlag	pBAD with *tisB* from +42 to +354, 3xFLAG tag inserted, Amp^{R}	[6]

Table S2. Oligodeoxyribonucleotides used in this study.

Name	Sequence (5' to 3')	Description
recB-KO-1	GCAGCAAACAATGCCCCTGATGAGTGAAAAGAATGAG TGAGCTCATATGAATATCCTCCTTAG	chr. Deletion of *recB* with *cat*
recB-KO-2	TGTGCTCCACAGCTTCCAGTAATTGCTTTTGCAATTTCAT CTAGAGCTAACTAACTTGTAGGCTG	chr. Deletion of *recB* with *cat*
recB-scr-1	CCTGAAGGCTGGAAAGTGTG	screening of chr. *recB* deletion
recB-scr-2	ACATCCAGCGGGCGTAG	screening of chr. *recB* deletion
ruvAB-KO-1	CATACAGCATTATCTTTGATTCATTACGCAGGAGCGTC ATGCTCATATGAATATCCTCCTTAG	chr. Deletion of *ruvAB* with *cat*
ruvAB-Ko-2	TATTGCCAGTGCCGGATGCGGCGCGAGCGACCAATCCG ACGCCTTTGAGTGAGCTGATAC	chr. Deletion of *ruvAB* with *cat*
ruvAB-scr-1	TCGCTGGATATCTATCCAGC	screening of chr. *ruvAB* deletion
ruvAB-scr-2	CGAACAGCGTCGCATCAG	screening of chr. *ruvAB* deletion
dinB-yfp-1	TGACCCGCAAATGGAAAGACAACTGGTGCTGGGATTA TGACCGAATTCAGAGAAAGAGGAG	*dinB-syfp2* fusion with *cat*
dinB-yfp-2	CTCATAATAATGCACACCAGAATATACATAATAGTATAC ACTAGAGCTAACTAACTTGTAGGCTG	*dinB-syfp2* fusion with *cat*
dinB-yfp-scr1	CAGCAAACCACCCAGGAG	screening of *dinB-syfp2* fusion
dinB-yfp-scr2	GAACCGCAACCGGTTGATC	screening of *dinB-syfp2* fusion
sulA_yfp_out	CAACTCTGGTTAACACCGC	screening of *sulA-syfp2* fusion
sYFP2_out	CGCGTCTTGTAGTTACCG	screening of *sulA-syfp2* fusion
topo-fw-Hind	TATAAAGCTTTGTTTTGGCGGATGAGAGA	pBAD amplification, HindIII
topo-rev-Eco	TATAGAATTCTGGAGAAACAGTAGAGAGTTG	pBAD amplification, EcoRI
syfp2-for-Eco	TATAGAATTCAGAGAAAGAGGAGAAATAC	*syfp2* amplification, EcoRI
syfp2-rev-Hind	TATAAAGCTTATTATTTATACAGCTCATCC	*syfp2* amplification, HindIII
yfp-1-alt	TGAGGGTGAAGGTGACGCAA	qRT-PCR against *syfp2*
yfp-2-alt	AACGCGCGAAACACTGAACG	qRT-PCR against *syfp2*
cysG-1-new	CGGACCGTGTTTTCGTCG	qRT-PCR against *cysG*
cysG-2-new	ACGGATCGCCACCTTTCA	qRT-PCR against *cysG*
hcaT-1	ATCCGTCCGACGATTCAG	qRT-PCR against *hcaT*
hcaT-2	CCGTAATAGGCCGCATGT	qRT-PCR against *hcaT*

Supporting references

1. Dörr, T.; Vulic, M.; Lewis, K. Ciprofloxacin causes persister formation by inducing the TisB toxin in *Escherichia coli*. *PLoS Biol* **2010**, *8*, e1000317.
2. Berghoff, B.A.; Hoekzema, M.; Aulbach, L.; Wagner, E.G.H. Two regulatory RNA elements affect TisB-dependent depolarization and persister formation. *Mol. Microbiol.* **2017**, *103*, 1020–1033.
3. Edelmann, D.; Oberpaul, M.; Schäberle, T.F.; Berghoff, B.A. Post-transcriptional deregulation of the *tisB/istR-1* toxin–antitoxin system promotes SOS-independent persister formation in *Escherichia coli*. *Environ. Microbiol. Rep.* **2021**, *13*, 159–168.
4. Datta, S.; Costantino, N.; Court, D.L. A set of recombineering plasmids for gram-negative bacteria. *Gene* **2006**, *379*, 109–115.
5. Vogel, J.; Argaman, L.; Wagner, E.G.H.; Altuvia, S. The small RNA IstR inhibits synthesis of an SOS-induced toxic peptide. *Curr. Biol.* **2004**, *14*, 2271–2276.
6. Unoson, C.; Wagner, E.G.H. A small SOS-induced toxin is targeted against the inner membrane in *Escherichia coli*. *Mol. Microbiol.* **2008**, *70*, 258–270.
